# Albumin-fatty acid interactions at monolayer interface

**DOI:** 10.1186/1556-276X-9-218

**Published:** 2014-05-07

**Authors:** Lai Ti Gew, Misni Misran

**Affiliations:** 1 Department of Biological Sciences, Faculty of Science and Technology, Sunway University, No. 5, Jalan Universiti, Bandar Sunway, Petaling Jaya, Selangor 46150, Malaysia; 2 Department of Chemistry, Faculty of Science, University of Malaya, Kuala Lumpur 50603, Malaysia

**Keywords:** Lipid-protein interaction, Protein, Stearic acid, Bovine serum albumin, Langmuir, Atomic force microscopy

## Abstract

The fluid mosaic model of Singer and Nicolson in 1972 shows how proteins are embedded in membranes. To elucidate the interactions between proteins and the surrounding lipids, stearic acid (SA) and bovine serum albumin (BSA) were used as lipid-protein components to mimic the normal membrane bilayer environment using the Langmuir-Blodgett technique. Surface pressure (*π*)-molecular area (*A*) isotherms were recorded for the SA monolayer in the presence of BSA on water. The mixed monolayer was successfully transferred onto an oxidized silicon wafer and imaged by tapping mode atomic force microscopy (AFM). Miscibility, compressibility and thermodynamic stability of the mixed system were examined. A large negative deviation of *A*_ex_, together with the minimum value of Δ*G*_ex_, was observed when the mole fraction of BSA (*X*_BSA_) was 0.8, indicating this to be the most stable mixture. In a compressibility analysis, *X*_BSA_ was observed at below 50 mN m^-1^, denoting a liquid-expanded phase and showing the occurrence of a strong interaction of SA with BSA molecules in this phase. AFM observations supported the quantitative data indicating that BSA was strongly attracted onto the membrane surface as predicted.

## Background

Immunoliposomes have been extensively developed for its potential as drug delivery carriers by attaching antibodies to the liposomal surface. Many *in vitro* studies using immunoliposomes in drug delivery to target cancer cells have greatly showed significant reduction in toxicities and improved therapeutic efficacy [1-4]. This promising approach can overcome challenges of targeting only the cancer and tumour cells that are often very similar in characteristics to the surrounding healthy tissue. Without the incorporation of targeting antibodies, liposomes would have to rely on nonspecific interactions with cell membranes. There are several proposed drug-loaded immunoliposome formulations that are used in drug delivery applications [5-8], but there is still scant knowledge on how liposomes interact with the antibodies they incorporate [9,10].

With the view of investigating interactions between liposomes and antibodies, we first set out to study the interactions between fatty acids and proteins by the Langmuir-Blodgett technique. Langmuir monolayers are widely used to model the biological membrane surface in studies to understand the structure and function of biological membranes and the protein-lipid interactions [11]. The way proteins assemble on the lipid bilayer, either partially or fully embedded, and their ensuing stability should be considered before any experiment on the incorporation of proteins in the membrane is performed [12,13]. In 1972, Singer and Nicolson made the important distinction between integral and peripheral membrane proteins in the fluid mosaic model of biological membranes [14]. Lipid-protein interactions that occur in the binary mixed system can be studied from data on miscibility, compressibility and thermodynamic stability from the isotherms obtained [15]. The analysed data would give an insight into intermolecular interactions between the lipid and protein, thereby providing useful information on the different ways proteins associate with cell membranes.

In our study, we used stearic acid (SA) to create a monolayer mimicking a half bilayer membrane, with various concentrations of bovine serum albumin (BSA) incorporated onto the monolayer. BSA is a globular protein that is highly water soluble and readily available at low cost. Its structural similarity to the human homologue makes it a widely studied protein [16]. To the best of our knowledge, the behaviour of BSA in a mixed lipid monolayer has not been studied in any great detail.

The outcome of this initial study would provide indicators for future work on the interactions of other globular proteins, including antibodies, in a mixed lipid monolayer.

## Methods

### Materials

A spreading solution of stearic acid (Sigma-Aldrich, Palo Alto, CA, USA) was prepared by dissolving it in analytical grade chloroform (Merck, Whitehouse Station, NJ, USA). Various concentrations of bovine serum albumin (Carl Roth GmbH, Karlsruhe, Germany) were prepared by dissolving in distilled water. Double-distilled water (processed by NANOpure Diamond Ultrapure Water System, Barnstead International, Dubuque, IA, USA) was used as the subphase throughout the study.

### Langmuir monolayer/mixed monolayer measurements

A computer-controlled Langmuir balance (KSV 5000, Langmuir System, Helsinki, Finland) equipped with symmetric barriers and Teflon trough (total area 60,720 mm^2^) was used to determine the surface pressure (*π*)-molecular area (*A*) isotherms. The surface pressure of the films was measured to an accuracy of ±0.1 mN m^-1^ using a flame-cleansed high-purity platinum metal Wilhelmy plate (19.62 mm × 10 mm) of 39.80-mm total length. The trough was filled with water (26°C ± 0.1°C) serving as the subphase. Solutions of SA and BSA were carefully transferred and spread randomly onto the subphase (water) using a Hamilton microsyringe (precision to 0.5 μl). The solutions were left for about 10 min to allow the solvent to evaporate before the *π*-*A* isotherms were measured. The films were compressed at a rate of 10 mm min^-1^.

### Y-type deposition of pure SA and SA/BSA on substrate

Silicon (100) wafers were cut into approximately 5 cm × 1 cm pieces and placed in a furnace (Carbolite, Watertown, WI, USA) for 8 h at 900°C to allow oxidation. The oxidized silicon wafer was clamped vertical to the subphase and immersed into the dipping well before spreading the monolayer material. After complete evaporation of the solvent, the floating layer was compressed at a rate of 10 mm min^-1^ to reach a target surface pressure of 20 mN m^-1^ and kept for 15 min to attain stability for deposition. The Y-type deposition of LB film was performed at the targeted pressure with a dipping speed of 10 mm min^-1^. All the transferred films were kept for a week in a dry, clean and closed container before atomic force microscopy (AFM) imaging.

### AFM imaging

High-resolution imaging of bilayers was obtained by AFM after transferring them from the air/water interface to a solid oxidized silicon substrate. Mixed bilayers from the Langmuir trough were transferred onto oxidized silicon substrates at the desired Wilhelmy pressure. Bilayers transferred to substrates were imaged using the NanoScopeIIIa scanning probe microscope controller (Veeco Instruments Inc., Plainview, NY, USA) in tapping mode under ambient conditions. Aluminum probes (Budget Sensors BS Multi 75Al, Innovative Solutions Bulgaria Ltd., Sofia, Bulgaria) were used. Resonance frequency of the probe was 75 kHz, and the force constant was 3 N m^-1^. Images in height mode were collected simultaneously with 256 × 256 points at a scanning rate of 1.0 Hz per line. A series of AFM images were taken from different perspectives.

## Results and discussion

### π*-*A *measurements and analyses*

#### π*-*A *isotherm*

**Figure 1:**
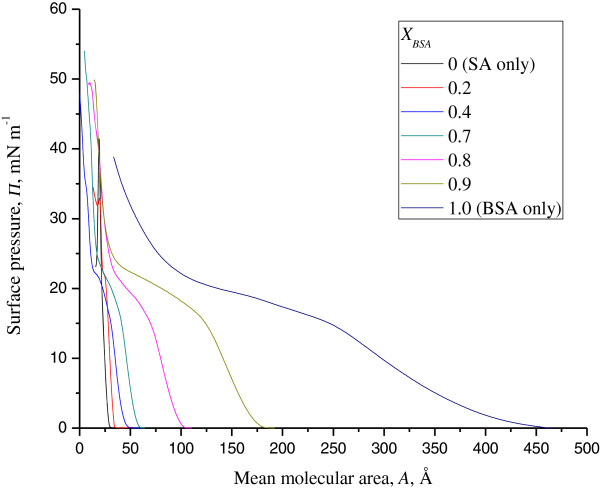
***π***-***A *isotherms for SA, mixtures of SA/BSA and BSA at the air/water interface at 26°C.**

Figure 1 shows a comparison between the surface pressure (*π*)-area (*A*) isotherms of the SA/BSA monolayer and the SA monolayer. The limiting area of the pure SA monolayer $A_{0}^{\text{SA}}$ was estimated to be 21 Å by extrapolating the straight portion of the *π*-*A* isotherm to zero surface pressure. The starting point of the straight portion at 20 to 25 mN m^-1^ represented a phase transition from liquid-condensed to the solid state (to be discussed later in the compressibility analysis). The SA monolayer collapsed at the surface pressure of 45 mN m^-1^.

When BSA was incorporated into the SA monolayer, the shape of the *π*-*A* isotherm gradually changed with increasing concentrations of BSA. All isotherms of the mixed systems shifted from the isotherm of pure SA to that of BSA as the mole ratio of BSA (*X*_BSA_) rose. This change showed that a mixed monolayer of SA/BSA was successfully formed, with more interactions between SA and BSA taking place as the concentration of BSA increased. A marked shift away from the isotherm of pure SA was observed at *X*_BSA_ = 0.8, 0.9 and 1.0 (the last value being pure BSA). There was no collapse pressure observed for *X*_BSA_ ≥ 0.9, suggesting that a stronger interaction occurred between SA and BSA with high concentrations of BSA in the mixed monolayer system.

#### Energetic stability of the mixed monolayers

The miscibility of the mixed monolayer components can be determined by calculating the mean molecular area *A*_12_.

For ideality of mixing, *A*_12_ is defined as

(1) $$A_{12}=X_{1}A_{1}+X_{2}A_{2}$$

where *A*_1_ and *A*_2_ are the mean molecular areas of single components at the same surface pressure and *X*_1_ and *X*_2_ are the mole fractions of components 1 and 2 in the mixed film. Quantitatively, these deviations can be described with the excess mean molecular area values too.

(2) $$A_{\text{ex}}=A_{12}–\left(A_{1}X_{1}+A_{2}X_{2}\right)$$

**Figure 2:**
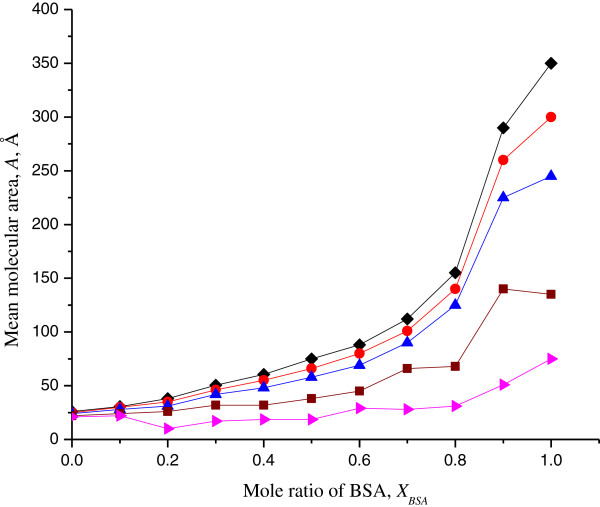
**Mean molecular area of SA/BSA monolayers vs *X***_**BSA **_**on pure water subphase at 26°C.** For discrete surface pressure of 5 mN m^**-**1^ (diamond), 10 mN m^**-**1^ (circle), 15 mN m^**-**1^ (triangle), 20 mN m^**-**1^ (square) and 25 mN m^**-**1^ (right-pointing triangle).

In Figure 2, the mean molecular area *A*_12_ is presented against *X*_SA_ at different surface pressures (5, 10, 15 and 20 mN m^-1^). A negative deviation from linearity was attributed to the miscibility of both components interacting with each other at the interface. The mean molecular area declined as the surface pressure increased. There were only slight deviations from ideality at 5 mN m^-1^, indicating immiscibility and weak interactions in a mixed monolayer. At 20 mN m^-1^, a marked negative deviation indicated strong attractions between the molecules in the mixed monolayer as compared with the interactions in their respective pure films. Large deviation observed at *X*_SA_ = 0.8 and 0.9 for the selected surface pressures showed a significant influence on the molecular packing and favourable interactions between molecules in the mixed monolayers.

The packing density of monolayers can be evaluated and analysed by the compression modulus *C*_*s*_^*-*1^, which is defined as [11,17]

(3) $$C_{s}^{-1}=-A\left(\frac{d\pi }{\text{dA}}\right)$$

*C*_*s*_^*-*1^ curves provide detailed information on phase transitions of SA/BSA monolayers. *C*_*s*_^*-*1^ can be classified into various phases, namely (a) liquid-expanded (LE) phase at surface pressure from 10 to 50 mN m^-1^, (b) liquid (L) phase from 50 to 100 mN m^-1^, (c) liquid-condensed (LC) phase from 100 to 250 mN m^-1^ and (d) solid (S) phase above 250 mN m^-1^. In this work, the compression moduli were obtained by numerical calculation of the first derivative from the isotherm data point using the OriginPro-8 program.

**Figure 3:**
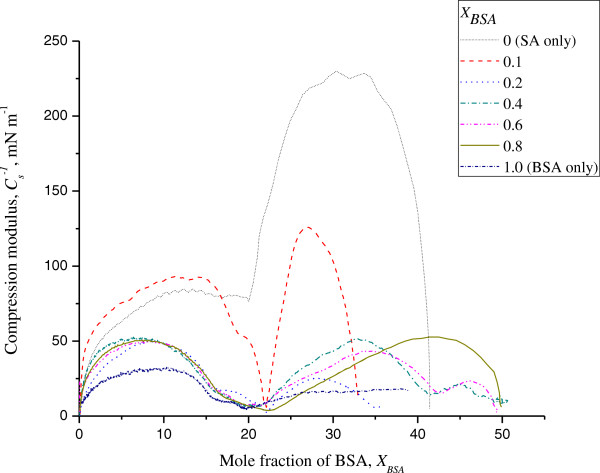
**Compressional modulus of SA/BSA monolayers vs surface pressure for discrete *X***_**BSA **_**on pure water subphase at 26°C.**

The significantly large value of compression modulus for the pure SA monolayer indicates its highly condensed phase (Figure 3). At 20 to 25 mN m^-1^, a change of its slope was observed, corresponding to the phase transition from the liquid-condensed to the solid state. *C*_*s*_^*-*1^ values of the mixed monolayers (*X*_BSA_ ≥ 0.2) were lower than that of the pure SA monolayer, indicating that the mixed monolayers were less condensed and more compressible than the pure monolayer. This is consistent with the plateau region existing in the *π*-*A* isotherm of every mixed system (Figure 1).

The isotherm of pure SA showed two distinct regions: the first one corresponding to the monolayer in its liquid-condensed (LC) phase and the second one of a solid (S) film that was characterized by higher *C*_*s*_^*-*1^ values. The values of *C*_*s*_^*-*1^ obtained that were relatively high at *X*_BSA_ = 0.1 are characteristic of a LC phase. The reason for this observation could be that at low concentrations of BSA, less lipid-protein interaction occurred in the mixed system.

At surface pressure 30 and 35 mN m^-1^, *C*_*s*_^*-*1^ was observed to be below 50 mN m^-1^ for the entire range of BSA mole ratios, from *X*_BSA_ ≥ 0.2 onwards, this being indicative of the formation of the LE phase. This implied that the incorporation of BSA into the SA monolayers reduced their condensation.

Molecular interactions can be expressed quantitatively in thermodynamic analysis. Total free energy of mixing Δ*G*_mix_ is defined by the following equation:

(4) $$\Delta G_{\text{mix}}=\Delta G_{\text{id}}+\Delta G_{\text{ex}}$$

where

(5) $$\Delta G_{\text{id}}=\text{RT}\;\left(X_{1}\text{ln}X_{1}+X_{2}\ln X_{2}\right),$$

and the excess free energy of mixing Δ*G*_ex_ can be calculated from *π*-*A* isotherms by [11,17]

(6) $$\Delta G_{\text{ex}}=\int_{0}^{\pi }\left[A_{12}-\left(X_{1}A_{1}+X_{2}A_{2}\right)\right]d\pi$$

where *A*_12_, *A*_1_ and *A*_2_ represent the area of the mixed system and respective areas of components as 1 and 2, respectively, and *π* is the surface pressure of the monolayer. If the monolayer is ideally mixed, Δ*G*_ex_ should be zero.

**Figure 4:**
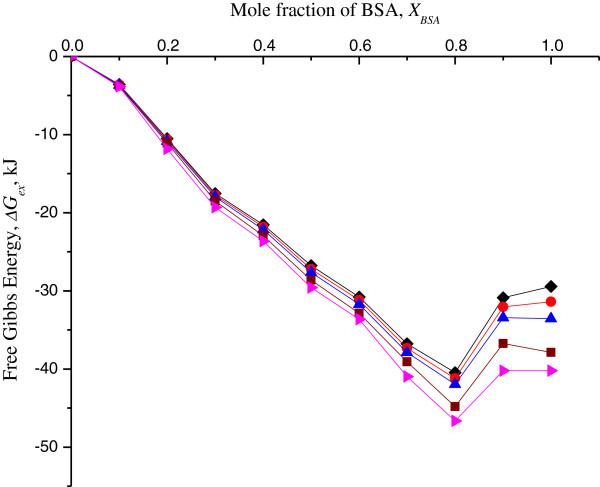
**Free excess energy Δ*G***_**ex **_**of SA/BSA monolayers vs *X***_**BSA **_**on pure water subphase at 26°C.** For discrete surface pressure of 5 mN m^**-**1^ (diamond), 10 mN m^**-**1^ (circle), 15 mN m^**-**1^ (triangle), 20 mN m^**-**1^ (square) and 25 mN m^**-**1^ (right-pointing triangle).

Negative values of Δ*G*_ex_ in the entire range of the monolayer composition indicated very strong attractions between molecules in the mixed system (Figure 4). The results showed that the binary SA/BSA mixed monolayers were thermodynamically stable. The most stable intermolecular interaction was observed at *X*_BSA_ = 0.8, at discrete surface pressures, suggesting that SA interacted strongly with BSA molecules and were miscible in the system. This observation was supported by the *A*_12_ and *C*_*s*_^*-*1^ measurements as discussed above.

Δ*G*_ex_ gradually decreased as the concentration of BSA rose. There was a slight recovery of Δ*G*_ex_ at *X*_BSA_ = 0.9. When the monolayer contained BSA only, Δ*G*_ex_ was almost similar to *X*_BSA_ = 0.9. This might be due to intermolecular repulsion occurring in the mixed monolayer system when the concentration of BSA was saturated in the system. The most compatible mixture of SA/BSA in a mixed monolayer was when *X*_BSA_ = 0.8.

### AFM observation of pure SA bilayer and SA/BSA mixed bilayer system

**Figure 5:**
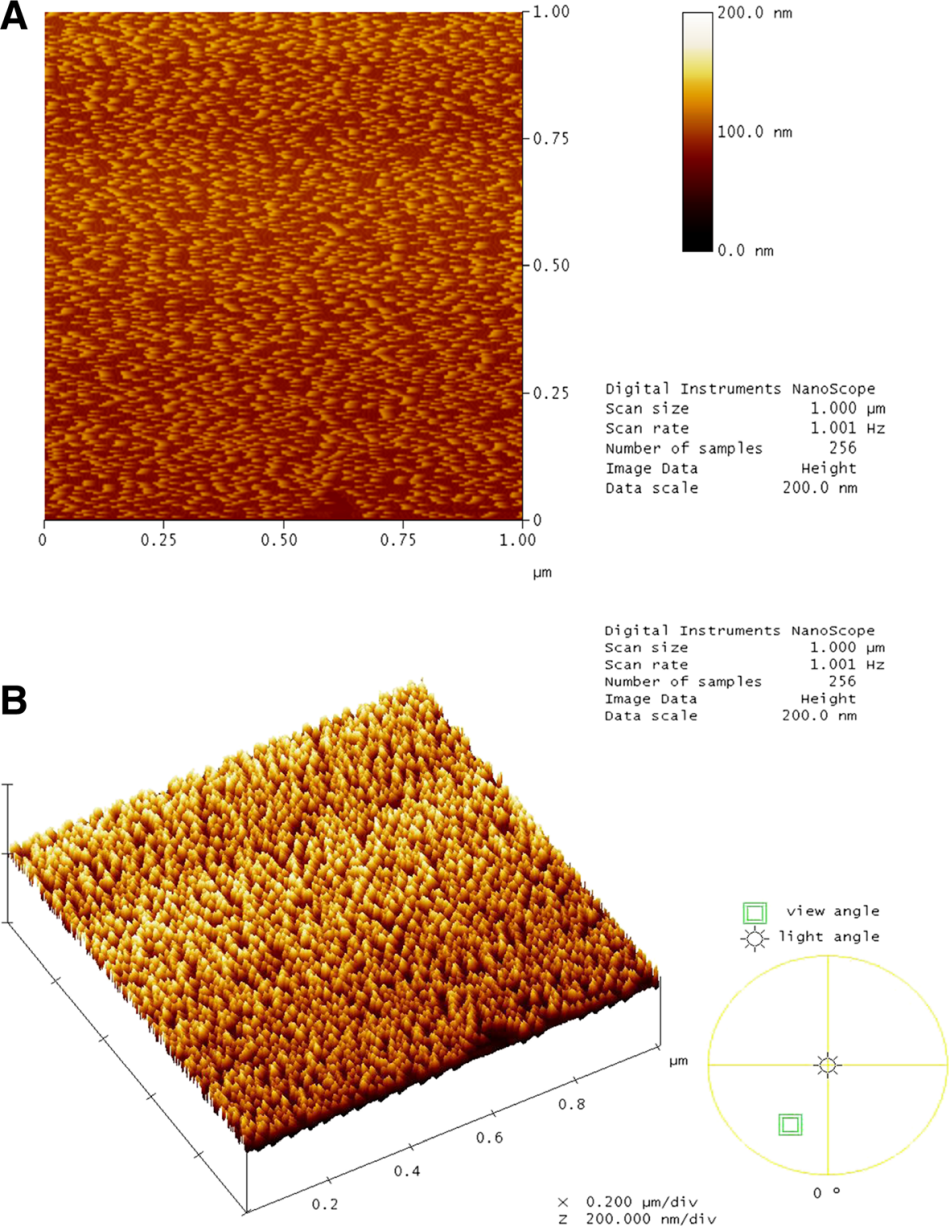
**AFM images of pure SA bilayer.** Deposited on oxidized silicon obtained in a 1.0 × 1.0 μm^2^ scan area and data scale of 200 nm. Similarly sized molecules that are arranged closely and orderly can be observed in the height top view **(A)** and from the 3D perspective shown in **(B)**. The SA bilayer arrangement is similar to the normal membrane bilayer.

**Figure 6:**
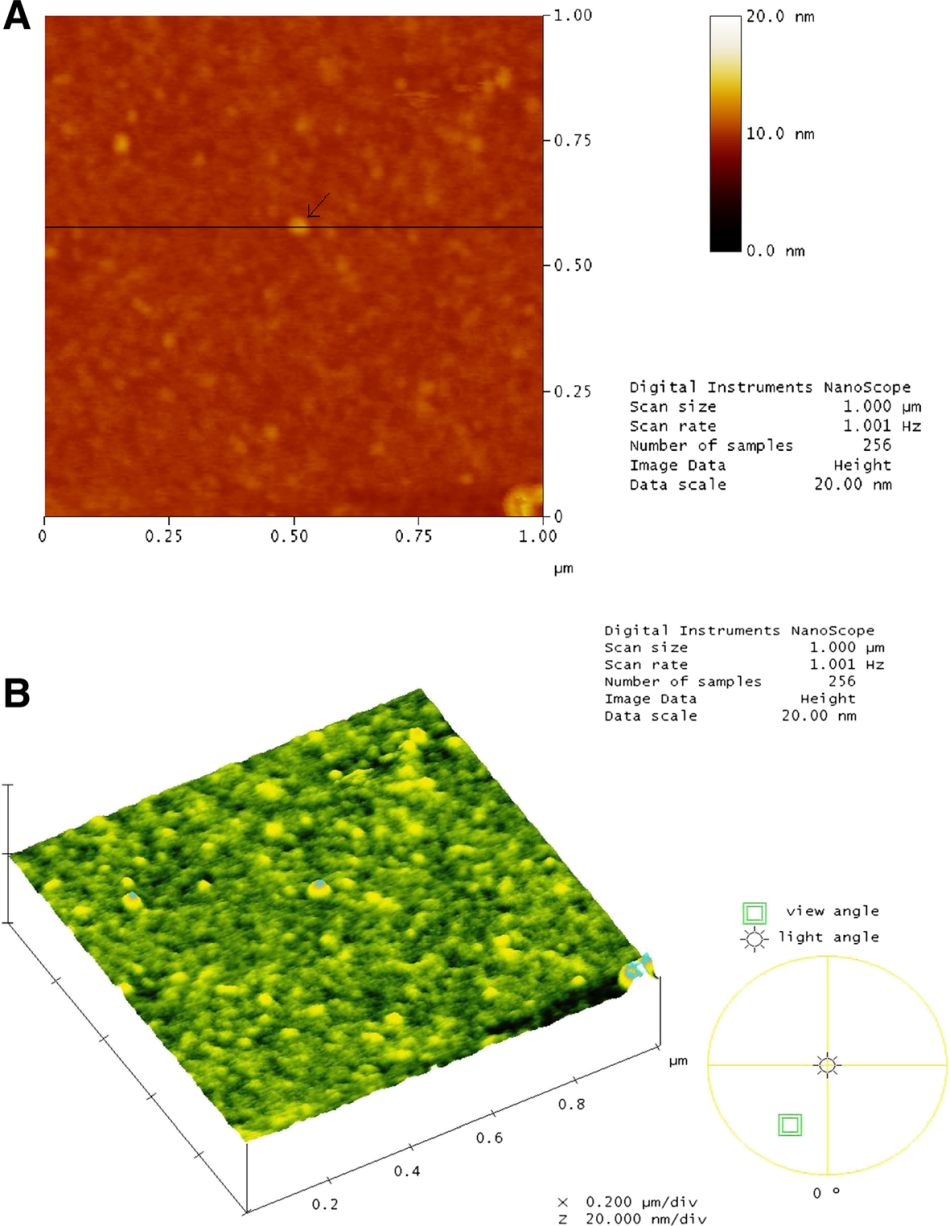
**AFM images of mixed SA**/**BSA bilayer (*X***_**BSA**_ **= 0.8).** Deposited on oxidized silicon obtained in a 1.0 × 1.0 μm^2^ scan area and data scale of 20 nm. The morphology of the binary system differs considerably from the images of pure SA in Figure 5. Irregularly sized small globular aggregations (in a brighter tone) can be observed randomly distributed in the height top view **(A)**. The 3D view in **(B)** shows the appearance of the globular protein, BSA, attracted strongly to SA that mimics a normal biological membrane. A cross section was drawn on a selected globular BSA incorporated on the membrane depicted in (A) to obtain more information of the height and width of BSA in the binary system. The height and width of this globular protein were found be to 2.781 and 54.688 nm, respectively.

AFM topography provides a morphological insight into the behaviour of the mixed system (Figures 5 and 6). AFM observations from this study supported our quantitative analysis which indicated that BSA was strongly attracted to the membrane surface as predicted from the theory.

## Conclusions

SA and BSA showed strong attraction as the concentration of BSA increased. The mixed monolayer was found to be most miscible at *X*_BSA_ = 0.8 as indicated by the negative Gibbs free excess energy. Analysis of the binary SA/BSA mixed monolayer confirms the spontaneous interaction between integral proteins and the lipids in accordance with the fluid mosaic model of Singer and Nicolson in 1972. The ensuing lipid bilayer with embedded proteins is thermodynamically stable, reflecting the situation in biological membranes.

## Competing interests

The authors declare that they have no competing interests.

## Authors' contributions

LTG participated in LB and AFM experimental work and drafted the manuscript. MM designed and coordinated the experimental study and helped draft the manuscript. Both authors read and approved the final manuscript.
